# ASO Author Reflections: Is There an Extra Benefit of Adding Neoadjuvant Radiotherapy to Chemotherapy in Patients with (Borderline) Resectable Pancreatic Cancer?

**DOI:** 10.1245/s10434-024-16829-x

**Published:** 2025-01-15

**Authors:** Won-Gun Yun, Jin-Young Jang

**Affiliations:** Department of Surgery and Cancer Research Institute, Seoul National University College of Medicine, Seoul, Republic of Korea

## Past

Over the past few years, several clinical trials have demonstrated that neoadjuvant chemotherapy can improve prognoses in patients with (borderline) resectable pancreatic cancer with major vessel invasion compared with upfront surgery.^1,2^ In addition to chemotherapy, the utility of radiotherapy is growing with the introduction of stereotactic body radiotherapy, which addresses the shortcomings of conventional radiotherapy such as long treatment course or low biologically effective dose. Therefore, it is time to evaluate the extra benefit of adding radiotherapy to chemotherapy, and the optimal time interval to undergo surgery following radiotherapy.

## Present

This study revealed that adding neoadjuvant radiotherapy to chemotherapy can improve postoperative overall survival, local control, and R0 resection rates compared with neoadjuvant chemotherapy alone in patients with (borderline) resectable pancreatic cancer.^3^ Additionally, the optimal interval to undergo surgery following radiotherapy was demonstrated to be within 4 weeks in terms of perioperative outcomes, including blood loss and postoperative pancreatic fistula.

## Future

Compared with previous studies, approximately two-thirds of this study cohort received neoadjuvant chemotherapy for more than 4 months and most patients received radiation doses > 50 Gy.^4^ These findings indicate that locoregional control can lead to improvement in prognosis under sufficient systemic control. In addition, the feasibility of more advanced radiotherapy techniques, including carbon ion radiotherapy or stereotactic magnetic resonance-guided adaptive radiotherapy, has been demonstrated. Future clinical trials evaluating the efficacy of neoadjuvant radiotherapy should be conducted in the near term, taking these factors into consideration.^5^
